# Identification of candidate genes involved in wax deposition in *Poa pratensis* by RNA-seq

**DOI:** 10.1186/s12864-016-2641-2

**Published:** 2016-04-29

**Authors:** Yu Ni, Na Guo, Qiuling Zhao, Yanjun Guo

**Affiliations:** College of Agronomy and Biotechnology, Southwest University, Chongqing, 400716 China

**Keywords:** Cuticular wax, *Poa pratensis*, Transcriptome, Unigenes, Very long chain fatty acids

## Abstract

**Background:**

The cuticular wax plays important roles in plant resistance to various biotic and abiotic stresses. Understanding the synthesis and secretion of cuticular waxes is necessary in utilizing cuticular waxes to improve crop productivity and plant ecological adaptation. Due to the lack of genomic resources, little genetic research on cuticular wax deposition has been focused on *Poa pratensis,* a perennial forage and turf grass species that is widely distributed under various habitats. In this study, we performed *de novo* transcriptome sequencing to explore differentially expressed genes between the leaf non-elongation zone (NEZm) and the emerged blade zone (EBZ) and to identify genes related to cuticular wax deposition.

**Results:**

A total of 77,707,414 high quality reads were obtained from llumina HiSeq 2500 platform, which were then assembled into 106,766 unigenes. Among them, 6019 unigenes showed significant differences in expression between NEZm and EBZ. In our assembled sequences, 3087 SSRs molecular markers were discovered. All the unigenes were searched against the NR, Swissprot, GO, COG, and KEGG databases using BLAST program for functional annotation. From 3156 unigenes with more expression in NEZm compared to EBZ, a number of unigenes involved in very long chain fatty acids (VLCFAs) and cuticular wax biosynthesis, transportation and regulation were identified. Several unigenes related to defense response and epidermal patterning were also found. Twelve putative genes involved in VLCFAs and cuticular wax biosynthesis were further analyzed for their expressions using qRT-PCR.

**Conclusions:**

The transcriptome of *P. pratensis* leaf was deep sequenced, *de novo* assembled and annotated, and the candidate genes potentially involved in VLCFAs and cuticular wax biosynthesis, secretion and regulation in *P. pratensis* were identified. This provides fundamental genetic resources in improving plant adaptation to abiotic and biotic stresses.

**Electronic supplementary material:**

The online version of this article (doi:10.1186/s12864-016-2641-2) contains supplementary material, which is available to authorized users.

## Background

Cuticular wax, as the major chemical component of plant cuticles, plays important physiological and ecological roles in the interactions between plants and their abiotic and biotic environments [1]. It has become clear that cuticular wax deposition could prevent water loss through leaf epidermis [2]; reduce water retention on leaf surface [3]; protect plant against enhanced ultraviolet radiation [4]; defend plant against pathogen attack [5]; and protect plant against air pollution and weathering [6]. The cuticular waxes are very long chain fatty acids (VLCFAs), consisting of hydrocarbons such as *n*-alkanes, primary alcohols, aliphatic ketones, *n*-alkanoic acids, as well as esters, aldehydes, and secondary alcohols [7]. Studies have shown that wax compositions differ in their functions and responses to abiotic and biotic stresses [8, 9]. Therefore, understanding the synthesis and transportation of each cuticular wax composition is necessary in utilizing cuticular waxes to improve crop productivity and plant ecological adaptation.

Wax biosynthesis begins with *de novo* C16 or C18 fatty acid biosynthesis in the plastid of epidermal cells. Then using the C16 or C18 acyl-CoA and malonylcoenzyme A as substrates, fatty acid elongase (FAE) complex performs a reiterative cycle of four reactions catalyzed by a β-ketoacyl-CoA synthase (KCS), a β-ketoacyl-CoA reductase (KCR), a β-hydroxyacyl-CoA dehydratase (HCD), and an enoyl-CoA reductase (ECR), to produce saturated VLCFAs with 24–36 carbon atoms. These VLCFAs are further modified to various wax molecules through two major pathways, the acyl-reduction and the decarbonylation pathways [10]. Using wax-deficient mutants, some genes encoding enzymes involved in these wax biosynthesis pathways have been cloned and characterized. The mutant loci in *Arabidopsis thaliana* are termed *eceriferum* (*cer*), and 21 independent *cer* loci have been identified in this plant model [11]. For example, *CER6/KCS6* and *CER10* have been identified for encoding KCS and ECR involved in VLCFAs biosynthesis [12, 13]. *CER8* encodes Long-chain Acyl-CoA Synthetase (LACS) catalyzing free fatty acids to CoA [14]. *CER3*, *CER1* and *MAH1* encode enzymes involved in the decarbonylation pathway which produces aldehydes, alkanes, secondary alcohols, and ketones [15, 16]. *CER4* and *WSD1* encode enzymes involved in the acyl reduction which forms primary alcohol and wax esters [17, 18]. *CER5*/*ABCG12* [19] and *ABCG11* [20], which belong to ATP-binding cassette (ABC) transporters, were reported to be required for *Arabidopsis* wax export. The identification of these wax-related genes helps understanding the production of cuticular wax and their functions.

Understanding the development of wax depositions during plant growth also provides an alternative way in screening cuticular wax genes. A microarray study on *Arabidopsis* by Costaglioli et al. (2005) showed that comparison of gene expression between (younger) wax synthesizing and (older) not wax-synthesizing shoot tissue was better suited for screening wax-related genes than comparison of expression between wild-type and *cer* mutant plants [21]. For monocotyledonous plants, Rhee et al. (1998) analyzed the wax deposition along the length of expanding leek (*Allium porrum* L.) and found that the level of total cuticular wax increased along the length of the leaf and the microsomal fatty acid elongation activities were induced within a defined and identifiable region of the expanding leek leaf [22]. In grasses, Richardson et al. (2007a) reported that cutin deposition in barley leaf epidermis occurred in parallel with cell elongation, whereas deposition of significant amounts of wax commenced as cells ceased to elongate [23]. Based on this finding, Richardson et al. (2007b) compared expression of candidate contig-sequences between leaf Elongation Zone, Non-Elongation Zone and Emerged Blade Zone, and identified candidate genes involved in wax deposition on barley leaves through a microarray approach [24].

*Poa pratensis* L, also known as Kentucky bluegrass, is a perennial forage and turf grass species that is well adapted to a wide range of mesic to moist habitats, and temperate to alpine conditions [25]. Pertierra et al. (2013) also reported that it was the longest surviving non-native vascular plant colony known in Antarctica from 1954 to 2012 [26]. The proportion of total cuticular wax present as alkanes was the highest in the alpine *Poa* species compared to low land *Poa* species [27], implying that the wide distribution of *P. pratensis* might be attributed to its ability in adjusting cuticular wax depositions. The RNA-Seq is a powerful, recently developed, high-throughput sequencing method, and provides new approaches to explore functional genes. In this study, we performed the pair end transcriptome sequencing of leaf non-elongation zone (NEZm) and the emerged blade zone (EBZ) of *P. pratensis* using the Illumina HiSeq platform. A total of 102,878,869 reads were obtained and assembled into 106,766 Unigenes. Thousands of potential simple sequence repeats (SSRs) molecular markers were discovered. About 6019 unigenes were differentially expressed between two leaf zones and a number of unigenes involved in cuticular wax deposition were identified. Twelve putative differentially expressed unigenes involved in cuticular wax biosynthesis were analyzed for their relative expression by further quantitative real-time PCR. The objective of this study was to provide a comprehensive molecular biology insight into the cuticular wax deposition in *P. pratensis* and to identify genes which are likely to be involved in wax deposition in *P. pratensis* leaves.

## Results

### Leaf cuticular wax

**Fig. 1:**
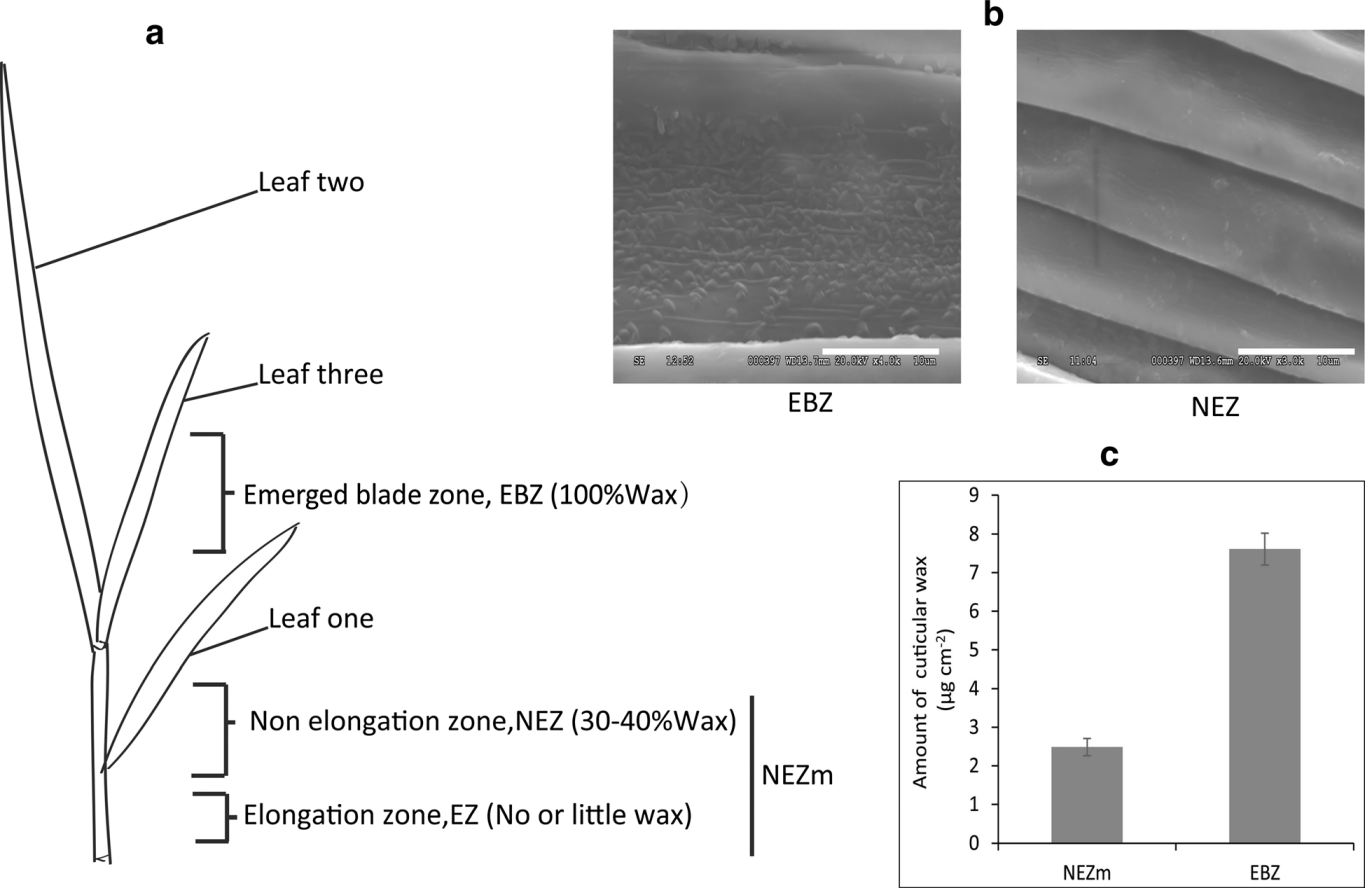
Leaf cuticular wax in non elongation zone (NEZm) and emerged blade zone (EBZ) of *Poa pratensis*. **a** Scheme of leaf regions which were analyzed by transcriptome sequencing and qRT-PCR; **b** wax crystal structure; **c** amount of total cuticular wax

Leaf three of *P. pratensis* was divided into mixed sample of non-elongation zone and elongation zone (NEZm) and emerged blade zone (EBZ) according to their positions (Fig. 1a). The NEZm was covered by sheath whereas the EBZ was fully expanded and exposed under air. Cuticular wax differentially deposited on the leaf zones of *P. pratensis.* Scanning electron microscope analysis showed that there was less wax crystalloids in NEZm than EBZ (Fig. 1b). The amount of total cuticular wax on EBZ was about three times than that in NEZm (Fig. 1c). In grasses, leaf cells divide and expand within the sheaths of older leaves, where the micro-environment differs from the open atmosphere. By the time epidermal cells are displaced into the atmosphere, they must have a functional cuticle to minimize uncontrolled water loss.

### *Poa Pratensis* transcriptome sequencing and *de novo* assembly

RNA samples from leaf NEZm and EBZ were subjected to Illumina HiSeq 2500 paired-end sequencing. In total, 102,878,869 reads with a total of 20,574,028,763 basepairs (20.57 Gb) were generated. After stringent quality assessment and data filtering, 77,707,414 reads (15.54 G) with 93.17 % Q30 bases (those with a base quality greater than 30) were selected as high quality reads for further analysis. An overview of the sequencing was presented in Table 1. All reads of sequencing have been deposited in the NCBIs Short Read Archive (accession number SRX1512962, SRX1512973, SRX1512974, and SRX1512975).

**Table 1:** Summary of the sequencing reads and reads after preprocessing

	*Poa pratensis*
Sequencing reads before preprocessing	
Total reads	102,878,869
Total bases	20,574,028,763
GC%	52.18
Q30%	80.39
Reads after trimming and preprocessing	
Total reads	77,707,414
Total bases	15,540,103,604
GC%	50.81
Q30%	93.17

Using the Trinity *de novo* assembly program [28], short read sequences were assembled into 8,032,387 contigs, of which there were 12,335 contigs coding for transcripts longer than 1 kb and 5435 contigs coding for transcripts longer than 2 kb. The contigs were subsequently subjected to cluster and assembly analysis. A total of 106,766 unigenes with N50 length of 1042 bp and mean length of 640.83 bp were obtained, among which 18,559 genes (17.38 %) were greater than 1 kb. An overview of the assembled contigs, transcripts and unigenes was presented in Table 2. These results demonstrated the effectiveness of Illumina pyrosequencing in rapidly capturing a large portion of the transcriptome.

**Table 2:** Summary of IIIumina transcriptome assembly for *Poa pratensis*

Length range	Total Length (Percentage)		
	Contigs	Transcripts	Unigenes
200–300	7,951,914(99.00 %)	65,336(23.44 %)	41,403(38.78 %)
300–500	40,282(0.50 %)	60,304(21.63 %)	29,234(27.38 %)
500–1000	22,421(0.28 %)	68,871(24.71 %)	17,570(16.46 %)
1000–2000	12,335(0.15 %)	60,743(21.79 %)	12,683(11.88 %)
≥2000	5435(0.07 %)	23,481(8.42 %)	5876(5.50 %)
Total number	8,032,387	278,735	106,766
Total length	466,581,232	239,460,551	68,419,199
N50 length	53	1313	1042
Mean length	58.09	859.10	640.83

Since there was no reference genome sequence for *P. Pratensis*, the *de novo* assembled transcriptome sequence by Trinity was regarded as a reference sequence. Bowtie software [29] was used to map all the clean reads to the *de novo* assembly transcriptome reference sequences and to qualify transcriptome by assigning to unigenes with the RSEM (RNA-Seq by Expectation Maximization) software [30]. The 59,429,338 clean reads (76.47 %) were successfully realigned to the reference sequence, showing that the quality of these assembled unigenes was sufficient to conduct the following analysis. Sequencing randomness and sequencing saturation assessment also demonstrated RNA integrity at transcript level (in Additional file 1: Figure S1).

**Fig. 2:**
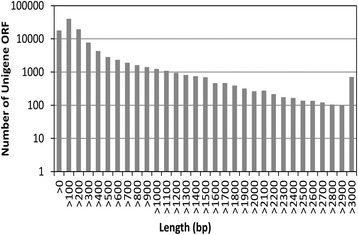
Length distribution of *Poa pratensis* unigene *ORF.* The y-axis indicates the number of unigene ORF, and the x-axis indicates the length of unigene ORF. Of the 106,766 unigenes, 106,000 unigenes were found to have predicted ORF by using the software Getorf. The minimum length was 57 bp and the maximum length was 10,376 bp. The largest number of ORF was in the range of 0–300 bp

Furthermore, 106,000 unigenes were found to have predicted ORF by using the software Getorf (http://emboss.sourceforge.net/apps/cvs/emboss/apps/getorf.html). The predicted ORFs ranged from 57 bp to 10,376 bp in length and N50 is 753 bp. The length distribution of the predicted ORFs was shown in Fig. 2.

### Frequency and distribution of SSRs in the *P. pratensis* leaf transcripts

SSRs markers are the most important molecular markers in plants and have been proven to be a valuable tool for various applications in genetics and plant breeding. In this study, 18,559 unigenes (more than 1 kb) were used to determine potential microsatellite motifs using MIcroSAtellite (MISA) software (http://pgrc.ipk-gatersleben.de/misa). In total 3087 SSRs were identified in leaf samples (Table 3). Tri-nucleotide repeats (1432, 46.39 %) were the most abundant SSR motif in leaf tissues followed by mono-nucleotide (1032, 33.43 %), di-nucleotide (412, 13.35 %), tetra-nucleotide (29, 0.94 %), penta-nucleotide (8, 0.26 %), and hexa-nucleotide (5, 0.16 %) motifs. The number of compound SSRs and uncertain compound SSRs were 157 (5.09 %) and 12 (0.39 %), respectively.

**Table 3:** Frequency of SSRs in *Poa pratensis*

Motif length	Repeat numbers							
	5	6	7	8	9	10	>10	Total (%)
perfect_SSR_1	-	-	-	-	-	588	444	1032 (33.43 %)
perfect_SSR_2	-	239	82	46	22	10	13	412 (13.35 %)
perfect_SSR_3	1042	280	97	13	-	-	-	1432 (46.39 %)
perfect_SSR_4	25	4	-	-	-	-	-	29 (0.94 %)
perfect_SSR_5	7	1	-	-	-	-	-	8 (0.26 %)
perfect_SSR_6	5	-	-	-	-	-	-	5 (0.16 %)
compound_SSR	-	-	-	-	-	-	-	157 (5.09 %)
compound_SSR*	-	-	-	-	-	-	-	12 (0.39 %)
Total SSR	1079	524	179	59	22	598	457	3087

Perfect_SSR_1, SSR_2, SSR_3, SSR_4, SSR_5, and SSR_6 represent mono-, di-, tri-, tetra-, penta-, and hexa-nucleotide repeat, respectively. Compound _SSR represents compound of two or more motifs. *uncertain SSR resulted from the compound

### Analysis of differentially expressed genes (DEGs)

**Fig. 3:**
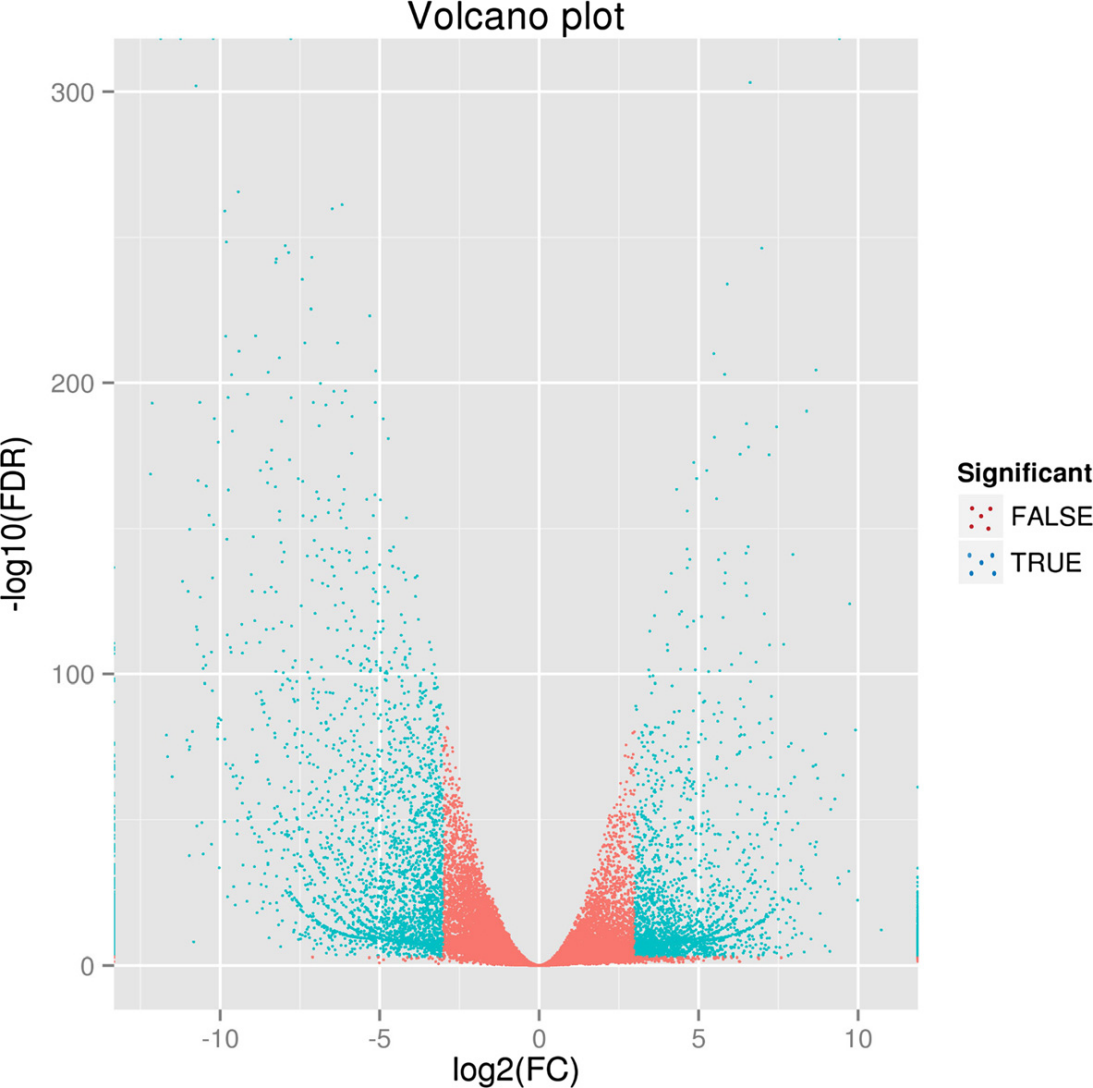
Volcano plot of differentially expressed genes. For the x-axis, the farther away from 0, the greater the difference of gene expression between the NEZm and EBZ. For the y-axis, the greater the value, the smaller the false positive rate, the more reliable the differentially expressed genes screened. FC: Fold change; FDR: False discovery rate; FALSE (red spot): insignificance; TRUE (blue spot): significance

The transcript abundance of each unigene was estimated by reads per kilobase of exon per million mapped reads (RPKM). Using software DESeq [31] and FDR ≤ 0.001 and ǀlog2FCǀ ≥ 8 as the criteria, 6019 unigenes showed significant differences in expression between the NEZm and the EBZ (Fig. 3), including both 2863 up-regulated unigenes and 3156 down-regulated unigenes in the EBZ library compared to those in the NEZm library.

### Functional annotation

For the validation and annotation of the assembled unigenes, all the assembled unigenes were searched against the NCBI non-redundant (NR), Swissprot, Gene ontology (GO), Clusters of Orthologous Groups (COG), and Kyoto Encyclopedia of Genes and Genomes (KEGG) databases using BLAST program with an E-value threshold of 10^-5^. Among 106,766 unigenes, 53,851 (50.44 %) and 32,710 (30.64 %) unigenes had significant matches in the NR and Swiss-Prot database, respectively (Table 4). Furthermore, GO, COG and KEGG annotations were applicable for 33.36, 12.45, and 7.91 % of unigenes, respectively.

**Table 4:** Functional annotation of the unigenes from *Poa pratensis*

Annotated databases	All sequence	> = 300 nt	> = 1000 nt
COG	13,296	11,188	6491
GO	35,619	26,932	12,428
KEGG	8449	6514	3311
Swiss-Prot	32,710	26,122	12,686
NR	53,851	39,903	16,597
All	54,219	40,083	16,623

**Fig. 4:**
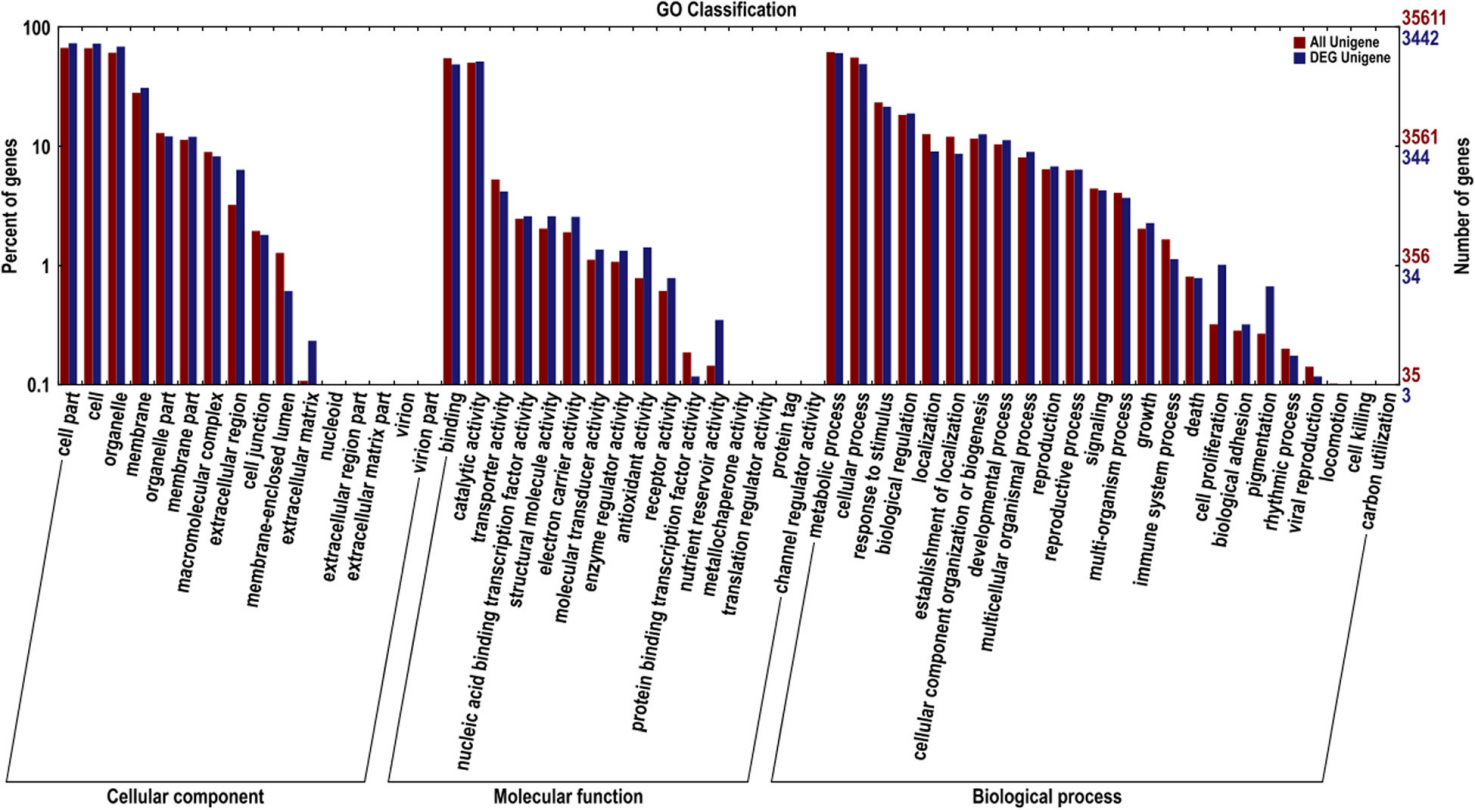
Histogram presentation of Gene Ontology classification. The results are summarized in three main categories, Cellular component, Molecular function, and Biological process. The left y-axis indicates the percentage of genes in a category, and the right y-axis indicates the number of genes in a category

There were 4782 DEGs annotated in NR database, among which 3442 DEGs were assigned with one or more GO terms (Fig. 4). In the biological process category, 2430 DEGs were classified into 24 GO classes, and “Metabolic process” and “Cellular process” were the most represented GO terms. In the cellular component category and the molecular function category, 1992 and 2545 DEGs fell into 16 GO classes, respectively. “Cell part”, “Cell” and “Organelle” were the most common terms in the cellular component category. Regarding molecular function, unigenes with binding and catalytic activity were highly represented (Fig. 4). Different enrichment trends between all unigenes and DEG unigenes were mainly observed in “Extracellular matrix”, “Extracellular region” and “Membrane-enclosed lumen” in the cellular component category, “Nutrient reservoir activity”, “antioxidant activity” and “protein binding transcription factor activity” in the molecular function category, and “Cell prolifereation” and “Pigmentation” in the biological process category, respectively (Fig. 4).

**Fig. 5:**
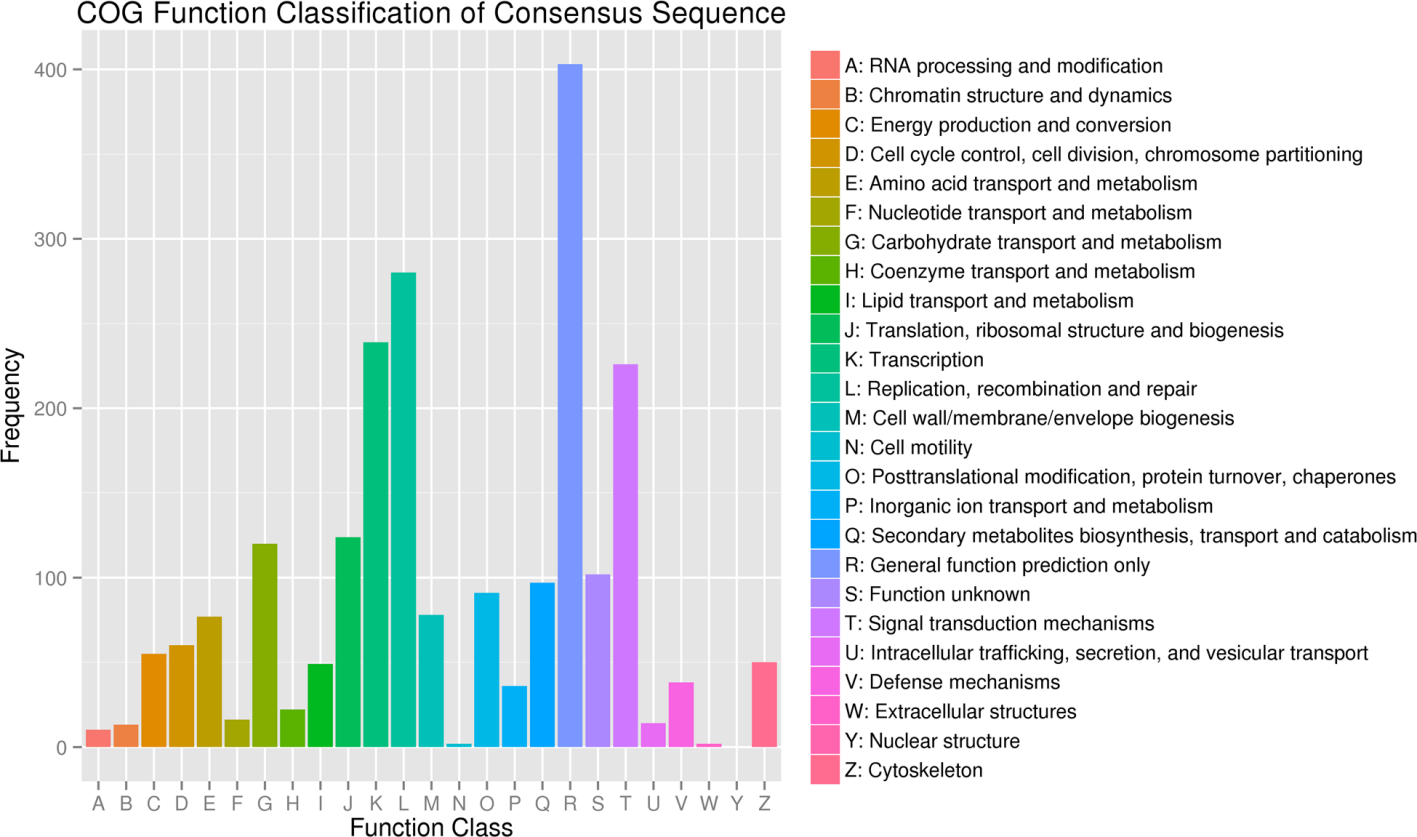
Histogram presentation of the COG classification of the differentially expression genes between leaf non-elongation zone and the emerged blade zone. In total, 1427 DEGs showing significant homology to the COGs database were grouped into at least 25 categories. The capital letters in x-axis indicate the COG categories as listed on the right and the y-axis indicates the number of DEGs in each category

DEG unigenes were also subjected to a search against the COG database for functional prediction and classification. Finally, 1427 DEGs could be assigned to COG classifications (Fig. 5). COG annotated putative proteins were functionally classified into at least 25 protein families involved in lipid transport and metabolism, transcription, signal transduction mechanisms, and so on. The cluster for general function prediction (403, 28.24 %) represented the largest group, followed by replication, recombination and repair (280, 19.62 %), transcription (239, 16.75 %), and signal transduction mechanisms (226, 15.84 %).

**Fig. 6:**
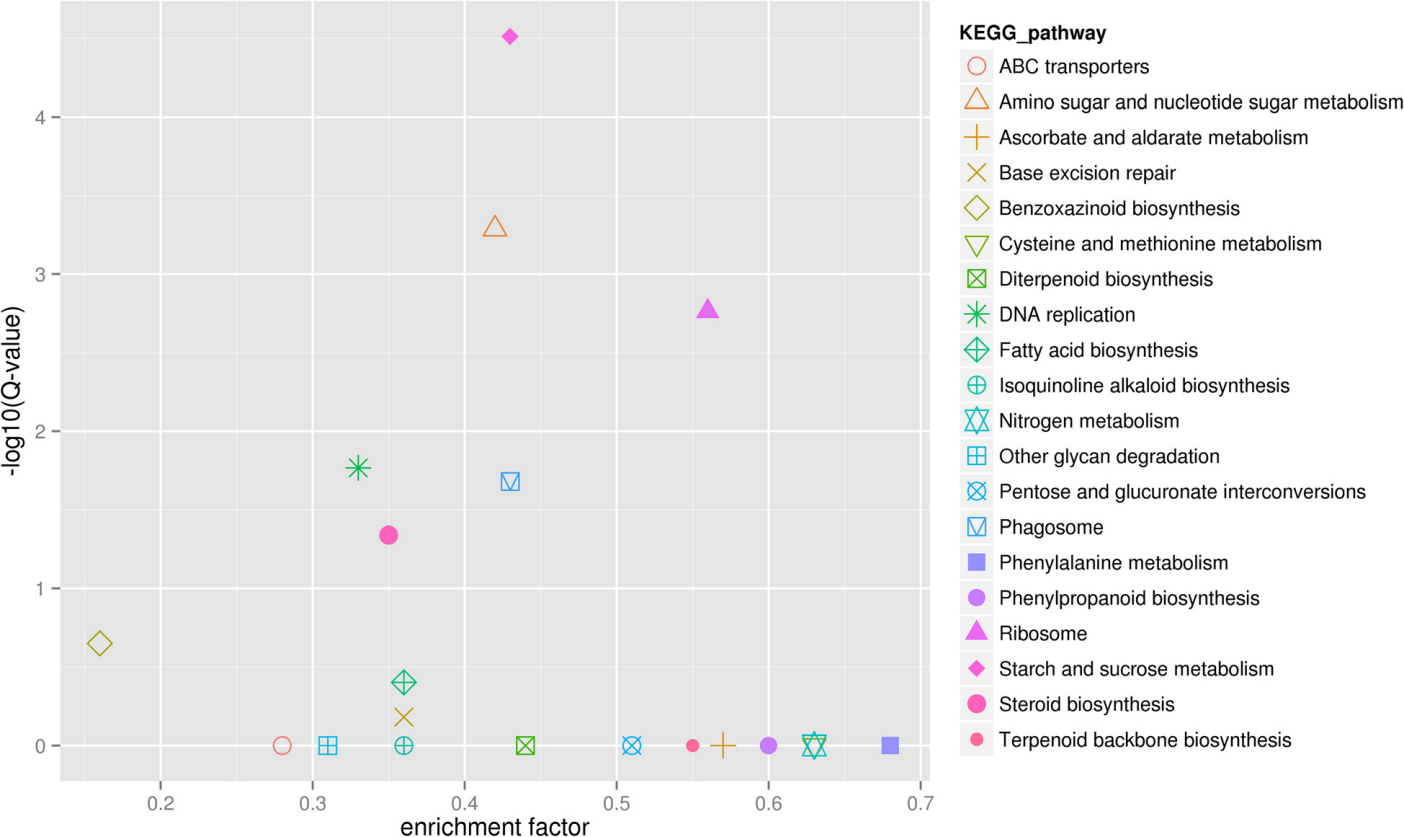
Scatter diagram of KEGG pathway enrichment of differentially expressed genes between leaf non-elongation zone and the emerged blade zone. Enrichment factor indicates the ratio of DEGs per pathway to all unigenes annotated in the pathway. Q-value is defined as an analog of the *p*-value that incorporates FDR-based multiple testing correction

To further analyze DEGs between leaf NEZm and the EBZ, all the DEG unigenes were analyzed in KEGG pathway database. As a result, 615 DEGs were found to have significant matches in the database and were assigned to 100 KEGG pathways (in Additional file 2: Table S1). The top 20 KEGG pathways with the most significant enrichment were shown in Fig. 6. It was also observed that there were 9 DEGs encoding enzymes that were involved in fatty acid biosynthesis.

### Differentially expressed genes involved in wax biosynthesis in *P. pratensis*

To identify the genes likely involved in wax biosynthesis in *P. pratensis*, we constructed a candidate fatty acid and wax deposition related gene set from down-regulated unigenes in the EBZ (non-active wax synthesizing) compared to the NEZm (active wax synthesizing) based on the GO, COG, KEGG, Swissprot, and NR annotation (in Additional file 2: Table S2). The selected unigene ID and annotation were listed in Additional file 2: Table S2. Three enzymes related to very-long-chain-fatty acid biosynthesis and six enzymes and proteins related to cuticular wax biosynthesis, secretion and regulation were identified. Putative proteins involved in defense response were also found (in Additional file 2: Table S3). In the most cases, more than one unigenes were assigned to the same enzyme or protein. Such unigenes might represent different fragments of a single transcript, different members of a gene family, or both.

### Quantitative real-time-PCR validation of the candidate DEGs involved in wax biosynthesis

**Fig. 7:**
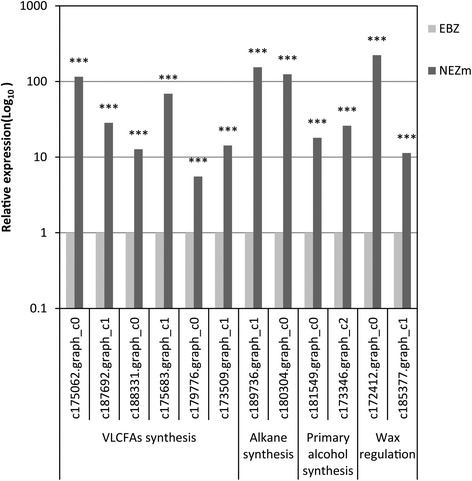
Validation of selected twelve up-regulated transcripts in the non-elongation zone as compared to the emerged blade zone involved in wax biosynthesis by qRT-PCR. EBZ and NEZm represent the emerged blade zone and the non-elongation zone, respectively. The relative gene expression levels as expressed by 2^-ΔΔCt^ were determined separately for each treatment as the mean ± SE. Asterisks indicate levels of significance of differential expression (*P* < 0.001)

To validate the responsible genes in the candidate gene set involved in wax deposition, twelve unigenes from the above set were selected and detected their expression profile between the NEZm and the EBZ by qRT-PCR. The results showed that twelve unigenes significantly up-regulated in the NEZm compared to the EBZ (Fig. 7), which were in consistent with the transcriptome data.

## Discussion

Transcriptome sequencing is widely applied in transcriptional and post-transcriptional regulation analysis of genes and global expression pattern analysis of complex genomes. In this study, a comparative *de novo* transcriptome analysis of leaf NEZm versus EBZ was conducted and 102,878,869 reads with a total of 20,574,028,763 bp (20.57 Gb) were generated by Illumina HiSeq 2500 paired-end sequencing. For plants without a genomic sequence, such short reads can be effectively assembled through the improvement of read length by the paired-end sequencing and the using of Trinity [28]. In total, 8,032,387 bp contigs, 278,735 bp transcripts and 106,766 bp unigenes were obtained in *P. pratensis,* respectively.

It was previously reported that wax deposition in barley started to be synthesized from the leaf NEZ wrapped by sheath [23]. In this study, transcript levels from the leaf NEZm and the EBZ were compared to obtain a list of significantly differentially expressed unigenes. Using DESeq software, which is suitable for screening differentially expressed genes in samples with biological replicates, and FDR ≤ 0.01 and ǀlog2FCǀ ≥ 2 as the criteria, 16,626 unigenes showed significant differences in expression between the NEZm and the EBZ. This number of DEGs was far beyond normal differential range. Considering the potential big difference between the two tissues and to increase the accuracy of the screening results, FDR ≤ 0.001 and ∣log2FC∣ ≥ 8 was used as adjusted criteria and finally 6019 DEGs were obtained. The functions of the DEGs by GO annotations were mainly related to “Extracellular matrix” and “Extracellular region” in the cellular components, and “Nutrient reservoir activity” in the molecular function category. These functions were in consistent with the vital importance of plant surface wax in protecting tissue from environmental stresses [9]. During cuticle deposition, a massive flux of lipids occurs from the sites of lipid synthesis in the plastid and the endoplasmic reticulum to the plant surface. The endoplasmic reticulum integrated with the membrane and plasmodesma in cellular components may play an important role in wax synthesis. Furthermore, DEGs were also mainly annotated with “Cell prolifereation”, attributing to different surface area expansion rates in NEZm and EBZ. This supports the phenomenon that leaf cells divide and expand within the sheaths of older leaves in grasses.

Based on Swissprot and NR annotation, several candidate genes involved in VLCFAs and cuticular wax biosynthesis pathway were obtained, such as *LACS*, *KCS*, *KCR*, *FAR*, and *CER1*. VLCFAs, as both wax component and wax precursor, was catalyzed by the elongase complexes, including KCS, KCR, HCD, and ECR. KCS is the first and committing step in VLCFAs biosynthesis and has broad substrate specificity [32]. Alcohol-forming fatty acyl-CoA reductase (FAR) and aldhydede carbonylase CER1 were identified catalyzing the production of primary alcohols and alkanes, respectively [15, 17]. LACS catalyzed free long-chain fatty acids to CoA [14]. In this study, *LACS*, *KCS*, *KCR*, *FAR* and *CER1* were expressed predominantly in the NEZm, the zone where significant wax deposition commences, supporting the previous results that younger shoot tissue was the active wax synthesis site [21]. In the developing barley leaf, wax deposition commences within the portion of the blade that is enclosed by sheaths of older leaves [33]. Changes in the micro-environment of the leaf from the NEZm to EBZ might alter the expression of the unigenes. However, five unigenes (c183078.graph_c0, c175984.graph_c0, c171931.graph_c1, c175102.graph_c0 and c171931.graph_c0) annotated as KCS and c185031.graph_c1 annotated as KCR were expressed higher in the EBZ compared to NEZm, suggesting the functional differentiation of KCS and KCR gene family members. Besides involving in cuticular wax synthesis, VLCFAs were also related to other physiological function such as involving in membrane lipids and sphingolipids [34]. Therefore, it is possible that only some KCS and KCR gene family members participate in wax production.

ABC transporter family was ubiquitously associated with transport across membranes of a broad range of molecules in prokaryotes and eukaryotes [35]. In this study, putative ABC transporter G, B and C families, and Lipid Transfer Proteins (LTPs) were significantly up-regulated in the NEZm compared to EBZ, indicating that these proteins might play an important role in *P. pratensis* wax secretion. It has been commonly proposed that ABC transporter G family member and LTPs were involved in wax trafficking from the endoplasmic reticulum to the plasma membrane and wax extracellular transportation, respectively [19, 36, 37].

The wax metabolic pathway is regulated by various transcription factors. One of these to be identified and characterized for its role in cuticle metabolism was SHN1/WIN1, a member of the *A. thaliana* ethylene-responsive AP2-domain transcription factor super family [38–40]. In this study, putative ethylene-responsive transcription factor, including *WIN1*and *WRI1*, were expressed mainly in the NEZm, which might activate wax deposition in younger tissue. The expression of *WIN1* was also related to defense response of plant. The overexpression of *SlSHN3,* an ortholog of the *Arabidopsis WIN/SHN3,* increased tomato resistance to *Botrytis cinerea*, whereas *SlSHN3*-RNAi plants were more sensitive to *B. cinerea* compared to wild-type plants [41]. The altered defense responses in *SlSHN3*-overexpressing plants were correlated with the cuticle permeability and the activation of pathogenesis-related gene *PR1a* and *AOS* [41]. Several defense response related genes, including *PR1*, were more expressed in NEZm compared to EBZ in this study, indicating that wax deposition might be linked with defense response. Garbay et al. [2007] reported that the amount of *PR1*-mRNA was not directly correlated to the amount of leaf epicuticular wax, but was rather correlated to the presence or absence of some particular lipid-constituents in the epicuticular wax layer [42]. Other transcription factors characterized for its role in cuticle metabolism belonged to MYB family. It is reported that MYB96 could promote drought tolerance and trigger disease resistance response by inducing *Arabidopsis* cuticular wax accumulation [43, 44]. The overexpression of *MYB30* in *Arabidopsis* stimulated the production of VLCFAs and waxes [45]. In this study, although no MYB96 and MYB30 were identified, fourteen unigenes annotated with Myb protein were found to have more expression in the NEZm compared to the EBZ, suggesting the potential role of MYB family in wax metabolism regulation.

Besides their primary role in stress response, cuticular waxes were also found to be involved in developmental processes, notably through tight connections with the epidermis morphology [46]. The more expression of epidermal patterning factor (EPF) in NEZm than that in EBZ supported the link between cuticular wax metabolism and the process of epidermal cell patterning (in Additional file 2: Table S4). In addition, putative bHLH transcription factors were also up-regulated in the NEZm (in Additional file 2: Table S5). This was in agreement with the function of bHLH, which was important in development or cell activity.

## Conclusions

In this study, a comparative transcriptome sequencing between leaf NEZm and EBZ in *P. pratensis* was performed using Illumina platform. In total, 77,707,414 clean reads were *de novo* assembled into 106,766 unigenes. All unigenes were then evaluated and functionally annotated by comparing with the existing protein databases, including NR, Swissprot, GO, COG, and KEGG database. Several candidate genes potentially involved in cuticular wax biosynthesis, transportation, regulation, development, and defense response, were identified. About 3087 SSR molecular markers were developed. The database will improve our understanding of the molecular mechanism of cuticular wax deposition in *P. pratensis* leaf and will provide the fundamental genetic resources in improving plant adaptation to abiotic and biotic stresses.

## Methods

### Plant material

*Poa pratensis* cv Nuglade plants were grown in pot (10 cm x 15 cm) filled with turfy soil in a growth chamber (15 °C /25 °C, RH 75 %). To make sure the plants grow well, 1/4 Hoagland solution were applied every 5 days. There were 70 pots with 15 plants in each pot. Forty 5 days after germination, most plants went into three leaf stage. According to the study of Richardson et al. (2007a), the leaf of barley was divided into three parts, elongation, non-elongation and emerged blade [23]. According to the protocol from Rymen et al. [47], for leaf three of *P. pratensis* in this study, the length of emerged blade, non-elongation and elongation parts reached about 10 cm, 1.5 cm and 0.5 cm, respectively. Since it was difficult to separate elongation zone and non-elongation zone clearly for RNA extraction and cuticular wax analysis separately, non-elongation and elongation zone (mainly non-elongation zone) were mixed together as one sample (NEZm) and were used for later analysis as well as emerged blade zone (EBZ) in this study. About 1 cm samples were collected from NEZm and EBZ of leaf three, separately, immersed in liquid nitrogen immediately, and then stored at−80 °C. About 200 plants from 20 pots were collected and bulked into one sample, approximately 0.1 g. In total two bulk samples were collected for RNA extraction from NEZm and EBZ, separately, as two biological replicates.

### Cuticular wax extraction and analysis

Samples from remaining 30 pots were used for wax extraction, with 10 pots as one replicate. The samples from NEZm and EBZ were separately dipped in 50 ml of chloroform containing 0.25 μg of hexadecane (Sigma) as internal standard for 30s at room temperature. The wax extractions were dried under nitrogen stream, derivated with 100 μl of BSTFA (N,O–Bis (trimethylsilyl) trifluoroacetamide) for 1 h at 80 °C, and the surplus BSTFA was evaporated under nitrogen. The extract was re-dissolved in 1 mL of hexane for wax analysis using GCMS–2010 (Shimadzu Technologies Co, Japan) equipped with a flame ionization detector (FID) as described by Kim et al. (2007) [48]. Peaks were assigned by comparing of their mass spectra with the mass spectral library, GCMS solution Software (Shimadzu, Japan). Amounts of cuticular waxes were expressed in μg/g.

### Scanning electron microscopy (SEM) analysis

Samples from NEZm and EBZ were collected, air dried, and then affixed to an aluminum stub with double sided adhesive tape. Stub was coated with gold and placed in the low-vacuum, variable-pressure chamber of the Hitachi S3500 Scanning electron microscopy and photographed with a digital camera with a digital camera at approximately 5000 magnification. Each sample replicated three times.

### RNA extraction, cDNA preparation and transcriptome sequencing

Total RNA was extracted from the EBZ and NEZm of *P. pratensis* leaf using TransZol kit (TransGen, China). DNA contamination was removed with RNase-free DNase I (Takara, China). RNA quality and quantity were assessed by absorption at 260 nm/280 nm, and gel electrophoresis. Briefly 2.5 μg of total RNA was enriched for Poly-A using NEBNext Poly (A) mRNA Magnetic Isolation Module (NEB, E7490). Transcriptome library for sequencing was constructed according to NEBNext mRNA Library Prep Master Mix Set for Illumina (NEB, E6110) and NEBNext Multiplex Oligos for Illumina (NEB, E7500). The prepared library was quantified using Library Quantification Kit-Illumina GA Universal (Kapa, KK4824) and validated for quality by running 1.8 % agarose gel electrophoresis. The library products were sequenced via Illumina HiSeq™2500 sequencer.

### Sequence data processing and *de novo* assembly

The raw reads generated by HiSeq2500 were cleaned by removing adaptor and primer sequences, reads in which the percentage of unknown bases (N) is greater than 5 % and low quality reads in which the percentage of the low quality bases was more than 20 %. Trinity [28] was used in *de novo* sequence assembly. First, Trinity combined the reads with a certain overlap length to form longer fragments, which were called contigs. Next, these reads were mapped back to contigs; with paired-end reads, Trinity was able to detect contigs from the same transcript and determine the distances between these contigs. Finally, Trinity connected these contigs into sequences that could not be extended on their end. Such sequences were defined as unigenes. Sequence saturation and distribution of reads on reference genes were analyzed to assess the overall sequencing quality. The ORFs were identified as the nucleotide sequence or as the protein translation provided by the “Getorf” software (http://emboss.sourceforge.net/apps/cvs/emboss/apps/getorf.html). The longest ORF was extracted for each unigene. The gene expression level was calculated using the RPKM method [49].

### Sequence annotation and functional characterization

The assembled sequences were annotated using BLASTX program against NCBI database and all unigenes were utilized for homology searches against protein databases such as NCBI Nr (http://www.ncbi.nlm.nih.gov/) and Swissprot (http://www.expasy.ch/sprot/). To further annotate the unigenes in this research, the Blast2GO program was used to get GO annotation according to molecular function, biological process and cellular component ontologies (http://www.geneontology.org/). Each annotated sequence may have more than one GO term, either assigned in the different GO categories or in the same category. Secondary metabolic Pathway assignments were performed according to the KEGG pathway database [50]. The unigenes sequences were also aligned to the COG database [51] to predict and classify functions. DESeq [31] was used to identify differentially expressed genes (DEGs) between the NEZm and EBZ. FDR (false discovery rate) ≤ 0.001 and Fold Change ≥ 8 were used as the threshold to judge the significance of gene expression difference. The annotation information from all the unigenes was extracted for the DEGs.

### Simple sequence repeats (SSRs) identification

All the unigenes with length more than 1 kb were used in a microsatellite program (MISA) (http:// pgrc.ipk-gatersleben.de/misa/misa/) for identification of potent SSR motifs. We searched for microsatellites from mononucleotide to hexa-nucleotide. Dinucleotide repeats of more than six times and tri-, tetra, penta and hexa- nucleotide repeats of more than five times were considered as the search criteria for SSRs in MISA script. Both perfect (i.e. contain a single repeat motif) and compound repeats (i.e. composed of two or more motifs separated by 100 bases) were identified.

### Quantitative real-time reverse transcription-PCR (qRT-PCR)

Twelve up-regulated unigenes in the NEZm with potential roles in cuticular wax deposition were chosen for validation using qRT-PCR. Total RNA was extracted from the EBZ and the NEZm of *P. pratensis* leaf using TransZol kit (TransGen, China). DNase-treated RNA was used to synthesize first strand cDNA by using SuperScript II reverse transcriptase (Invitrogen, China). The gene names and primers used for qRT-PCR are liste in Additional file 2: Table S6. The quantitative reaction was performed on the CFX96 Real-Time PCR Detection System (Bio-Rad) using the SYBR Premix Ex TaqII(Takara, China). qRT-PCRs were performed as follows: 95 °C for 30 s, 40 cycles of 95 °C 5 s, 59 °C 30 s, and 72 °C 15 s. CFX Manager software (Bio-Rad) was used for data analysis. Expression levels of the selected unigenes were normalized to that of Elongation factor 1 (eEF1-a), an internal reference gene [52]. The relative expression levels of target genes were calculated with the 2^-ΔΔCt^ method [53]. All the experiments were repeated using three biological and three technical replicates and the data were analyzed statistically.

### Availability of data and material

The data sets supporting the results of this article are included within the article and its additional files.

## Additional files

Additional file 1: Figure S1. A, Randomness test of cDNA fragments; B, Sequencing saturation analysis. T1 and T2 represent NEZm; T3 and T4 represent EBZ. (PDF 290 kb) Additional file 2: Table S1. List of DEG unigenes between NEZm and EBZ related to KEGG pathway. **Table S2**. Candidate genes involved in fatty acids and cuticular wax biosynthesis in *P. pratensis* leaf. **Table S3.** List of unigenes involved in defense response that is differentially expressed in EBZ versus NEZm. **Table S4**. Putative epidermal patterning factor (EPF) differentially expressed between NEZm and EBZ. **Table S5.** Putative bHLH transcription factor differentially expressed between NEZm and EBZ. **Table S6.** The primers used in quantitative real-time PCR analysis. (XLS 118 kb)

## Abbreviations

ABC: ATP-binding cassette
COG: Clusters of Orthologous Groups
EBZ: emerged blade zone
ECR: enoyl-CoA reductase
FAE: fatty acid elongase
FAR: fatty acyl-CoA reductase
GO: Gene ontology
HCD: β-hydroxyacyl-CoA dehydratase
KCR: β-ketoacyl-CoA reductase
KCS: β-ketoacyl-CoA synthase
KEGG: Kyoto Encyclopedia of Genes and Genomes
MISA: MIcroSAtellite
NEZm: mixed sample of non-elongation zone and elongation zone
NR: non-redundant
qRT-PCR: quantificational real-time polymerase chain reaction
RSEM: RNA-Seq by Expectation Maximization
SSRs: simple sequence repeats
VLCFAs: very long chain fatty acids
