# Supplementary material for: Identification of candidate genes involved in wax deposition in Poa pratensis by RNA-seq
Source: BMC Genomics. 2016 Apr 29;17:314. doi: 10.1186/s12864-016-2641-2 (PMC4850629; doi:10.1186/s12864-016-2641-2)
Supplement: Additional file 1: Figure S1. — A, Randomness test of cDNA fragments; B, Sequencing saturation analysis. T1 and T2 represent NEZm; T3 and T4 represent EBZ. (PDF 290 kb) [file 12864_2016_2641_MOESM1_ESM.pdf]

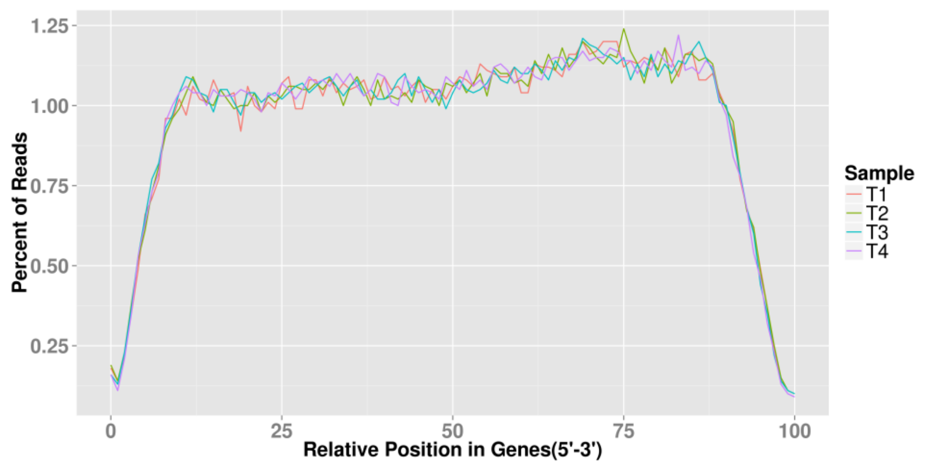


A

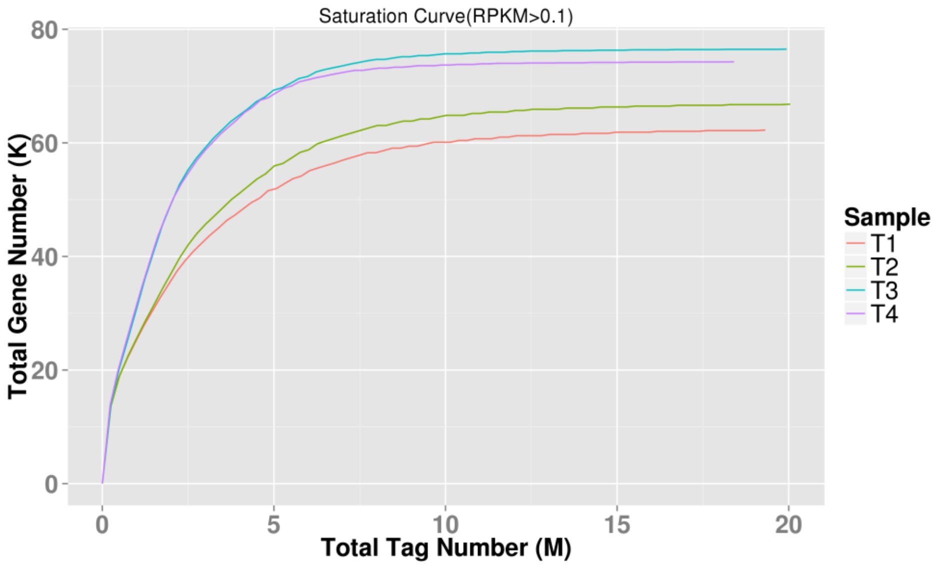


B

**Additional file 1: Figure S1. A, Randomness test of cDNA fragments; B, Sequencing saturation analysis.** T1 and T2 represent NEZ; T3 and T4 represent EBZ.
